# Human synovial mesenchymal stem cells show time-dependent morphological changes and increased adhesion to degenerated porcine cartilage

**DOI:** 10.1038/s41598-022-20386-2

**Published:** 2022-10-05

**Authors:** Takahiro Tanimoto, Kentaro Endo, Yuriko Sakamaki, Nobutake Ozeki, Hisako Katano, Mitsuru Mizuno, Hideyuki Koga, Ichiro Sekiya

**Affiliations:** 1 Center for Stem Cell and Regenerative Medicine, Tokyo Medical and Dental University, 1-5-45 Yushima, Bunkyo-ku, Tokyo 113-8510 Japan; 2 Research Core, Tokyo Medical and Dental University, Tokyo, Japan; 3 Department of Joint Surgery and Sports Medicine, Graduate School of Medical and Dental Sciences, Tokyo Medical and Dental University, Tokyo, Japan

**Keywords:** Regenerative medicine, Stem-cell biotechnology

## Abstract

The possibility that mesenchymal stem cells (MSCs) can adhere to partial defects or degenerative areas in cartilage remains to be established. The purposes of the present study were to verify the adhesion of synovial MSCs to degenerated cartilage, the time course of that adhesion, and the morphological changes that MSCs might undergo during the adhesion process. The surface of pig cartilage was abraded, and a human synovial MSC suspension was placed on the abraded surface. The proportion/number of MSCs that adhered to the cartilage was quantified by counting non-adhered MSCs, measuring the fluorescence intensity of DiI-labeled MSCs, and scanning electron microscopy (SEM) observations. The presence of microspikes or pseudopodia on the MSCs that adhered to the cartilage was also evaluated. SEM confirmed the adhesion of synovial MSCs to degenerated cartilage. The three independent quantification methods confirmed increases in the proportion/number of adhered MSCs within 10 s of placement and over time up to 24 h. The MSCs that adhered at 10 s had a high proportion of microspikes, whereas those that adhered after 1 h had that of pseudopodia. MSCs showed time-dependent morphological changes and increased adhesion to degenerated cartilage after placement of the human synovial MSC suspension.

## Introduction

Mesenchymal stem cells (MSCs) are defined by their origin in mesenchymal tissue and by their functional capacity to self-renew and differentiate into various progeny types^1^. In regenerative medicine, MSCs are appreciated for their simplicity of preparation and low risk of tumorigenesis after transplantation^2^. MSCs can be obtained from several types of mesenchymal tissues, and show some features in common as well as properties specific to their origins. MSCs derived from the synovium (synovial MSCs) are attractive for cartilage regenerative medicine because of their high chondrogenic potential^3^.

Cartilage does not spontaneously regenerate after trauma, as it has no blood vessels and sparse cellular components^4^. Autologous chondrocyte implantation is one representative treatment option for repairing cartilage defects^5,6^. While this type of implantation has the advantage of regenerating hyaline cartilage in cartilage defects, it has the disadvantage that the number of chondrocytes available for transplantation is limited due to the small amount of cartilage tissue that can be harvested and the low chondrocyte proliferative capacity. The use of MSCs can overcome this problem.

Cartilage defects can be divided into full-thickness cartilage defects and partial-thickness cartilage defects. A full-thickness cartilage defect is generally one where the injured superficial cartilage extends to the deep calcified cartilage, whereas a partial-thickness cartilage defect is limited to superficial cartilage injury^7,8^. Successful cell therapy depends primarily on the adhesion of the transplanted cells to the target site. Transplantation of chondrocytes or MSCs is not considered suitable for partial-thickness cartilage defects since the remaining extracellular matrix of articular cartilage does not provide a favorable environment for the adhesion of the transplanted cells^9–12^. By contrast, in a full thickness cartilage defect, the exposed subchondral bone provides a suitable substrate for cell adhesion and facilitates the role of the transplanted cells in the repair^13–15^. Therefore, chondrocytes or MSCs are usually transplanted after removal of the entire layer of articular cartilage from the injury site. However, in partial-thickness cartilage defects, the deeper cartilage is not severely damaged. Intentional removal of this cartilage results in deeper and wider cartilage defects, which eventually worsen symptoms. Therefore, the establishment of minimally invasive treatments for partial-thickness cartilage defects is desirable.

Cartilage loss is a serious consequence of osteoarthritis (OA) of the knee, a disease that is primarily caused by aging and that impairs walking ability^16^. The initial structural change observed in the cartilage of OA knees is a degenerative alteration of the superficial layer^17^. A growing number of reports now indicate that intraarticular injections of MSCs can reduce knee pain in patients with OA^13,14^. This is presumably because most of the injected MSCs become engrafted to the synovium, where they produce trophic factors^18^. Some reports also indicate that a fraction of the MSCs injected into OA knees adhere to the damaged cartilage^15,19,20^.

Techniques to attach more MSCs to damaged or degenerated cartilage simply and quickly may lead an improvement in the treatment of cartilage injury and OA with MSCs. The mechanisms how MSCs adhere to damaged or degenerated cartilage is not fully elucidated at this time. MSC therapy for traumatic cartilage injury and OA could lead to less invasive and simpler treatments if MSCs can be proven to adhere to the cartilage with partial thickness defect or degeneration. The purposes of the present study were to clarify whether synovial MSCs can adhere to degenerated cartilage, the time course and extent of their adhesion, and the morphological changes that MSCs might undergo during the adhesion process.

## Results

### Characteristics of human synovial MSCs

**Figure 1:**
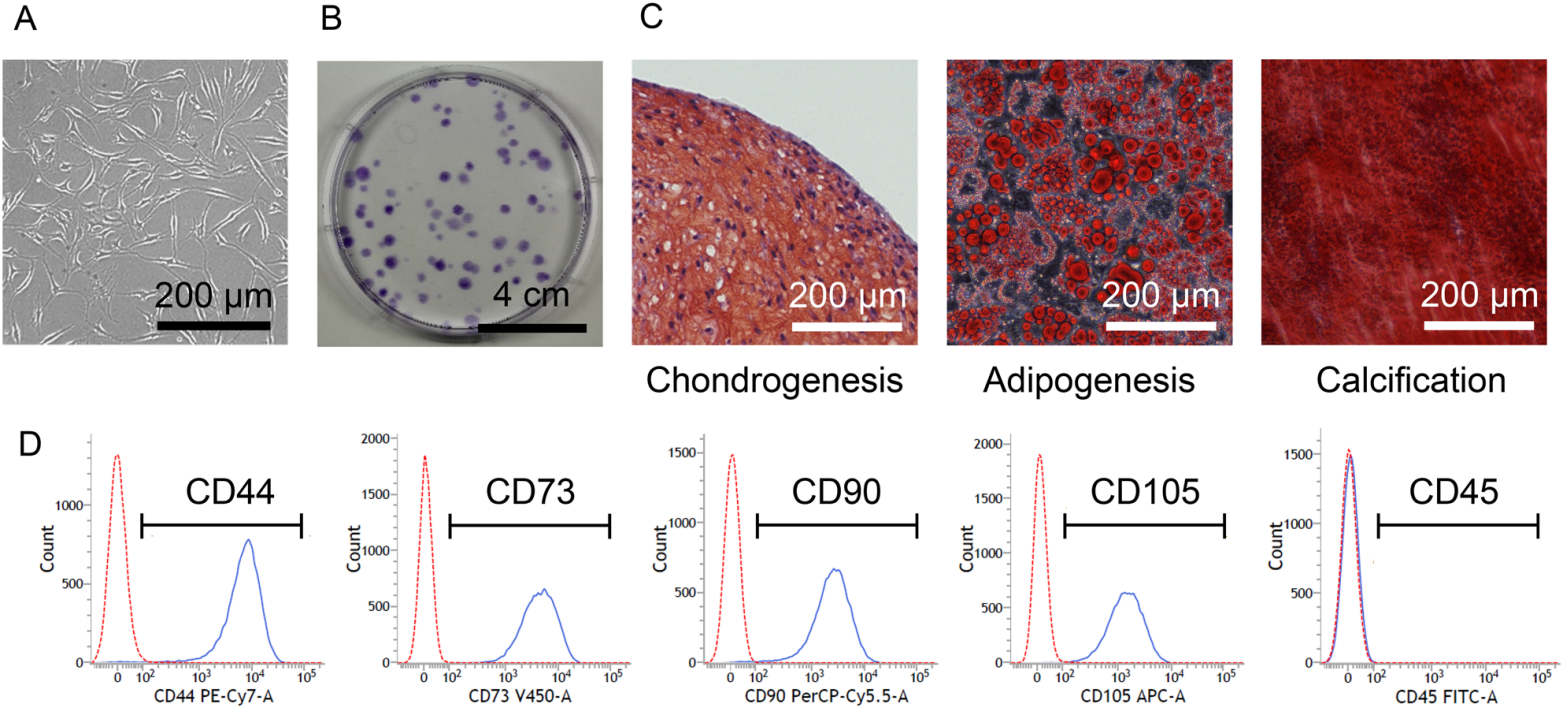
Characteristics of human synovial mesenchymal stem cells. (**A**) Cell morphology. (**B**) Colony formation ability. (**C**) Multidifferentiation ability. (**D**) Surface antigen expression. The indicated surface antigen is shown in blue and the isotype control is shown in red.

The cells were spindle-shaped (Fig. 1A) and formed colonies after 14 days of culture (Fig. 1B). They differentiated into cartilage that stained with safranin O, adipocytes that stained with oil red O, and calcified tissue that stained with alizarin red (Fig. 1C). They expressed CD 44, 73, 90, and 105, but not CD45 (Fig. 1D).

### Abraded porcine cartilage

**Figure 2:**
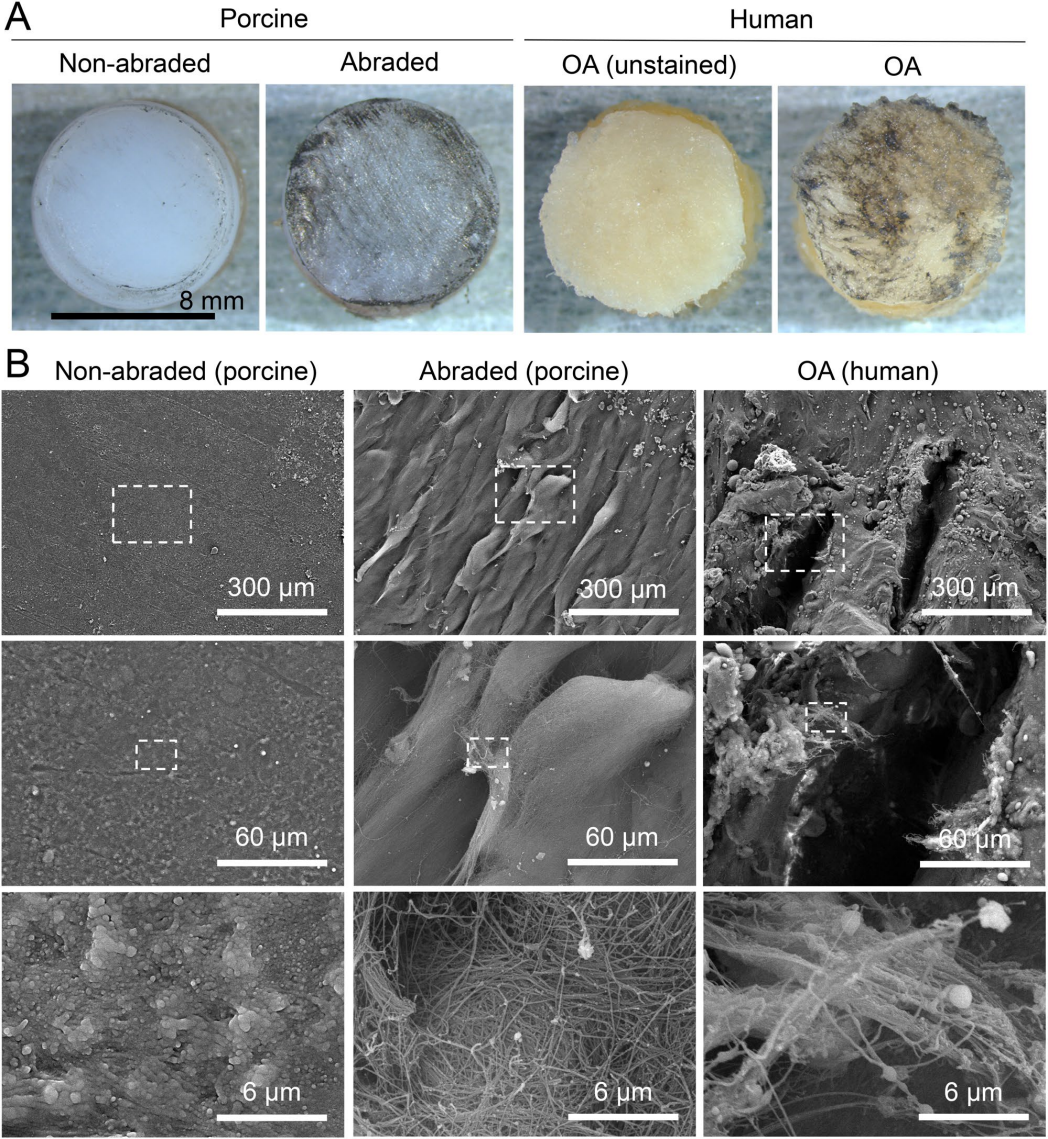
Surfaces of abraded porcine cartilage and human OA cartilage. (**A**) Gross appearances of the cartilage surfaces after staining with India ink. (**B**) Scanning electron microscopy images of the cartilage surfaces.

Abrasion of the surface of porcine cartilage induced similar India ink staining to that observed with human OA cartilage (Fig. 2A). SEM images showed a sawtooth-like structure with a width of 100 μm on the abraded surface, whereas the non-abraded cartilage retained a smooth surface (Fig. 2B). Increased magnification revealed partial fibrillation of the surface of the abraded cartilage. Further magnification revealed frayed and intertwined collagen fibers that resembled the features of human OA cartilage.

### Quantification of numbers of adhered MSCs

**Figure 3:**
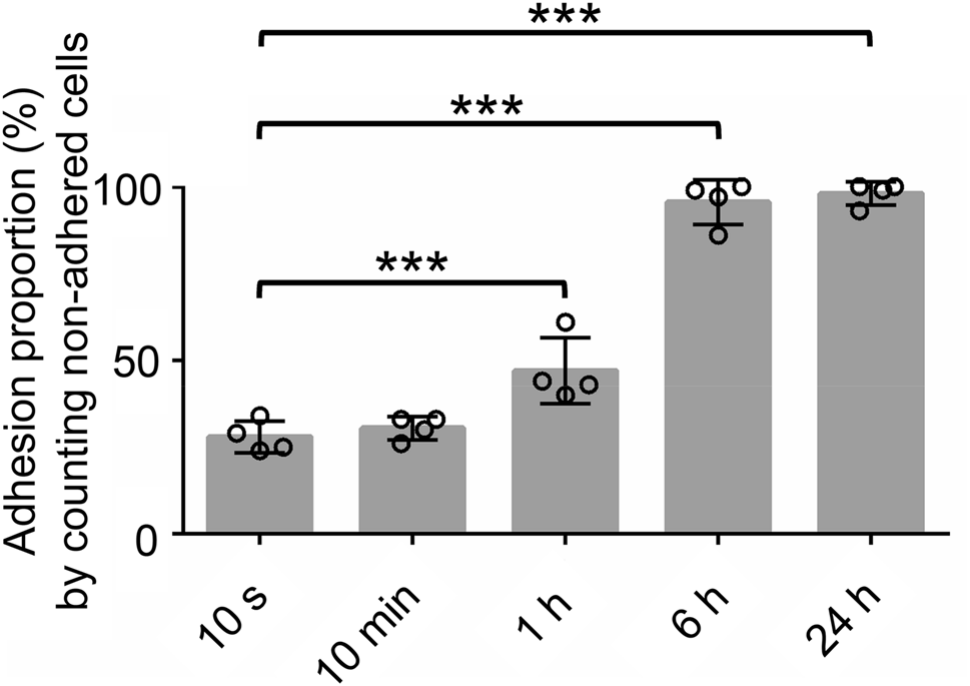
Quantification of numbers of adhered MSCs by counting non-adhered cells. An MSC suspension was placed on the abraded porcine cartilage, the cartilage was washed with PBS after a specified time, and the number of MSCs in the PBS that did not adhere was counted. The number of MSCs that adhered was calculated by subtracting the number of MSCs that did not adhere from the total number of MSCs that had been applied and this value was used to calculate the adhesion proportion. The number of cylindrical cartilage pieces obtained per MSC donor was 5 or 6. This experiment was repeated four times independently and plotted. Data are expressed as the average ± standard deviation (***p < 0.001).

We quantified the numbers of synovial MSCs that adhered to abraded porcine cartilage by three methods. As the first method, the proportion of adhered MSCs was calculated by counting the non-adhered MSCs retrieved after washing with PBS. This proportion was 28% at 10 s and at 10 min, but increased significantly to 50% at 1 h and further increased to 90% at 6 h, with no further changes at 24 h (Fig. 3).

**Figure 4:**
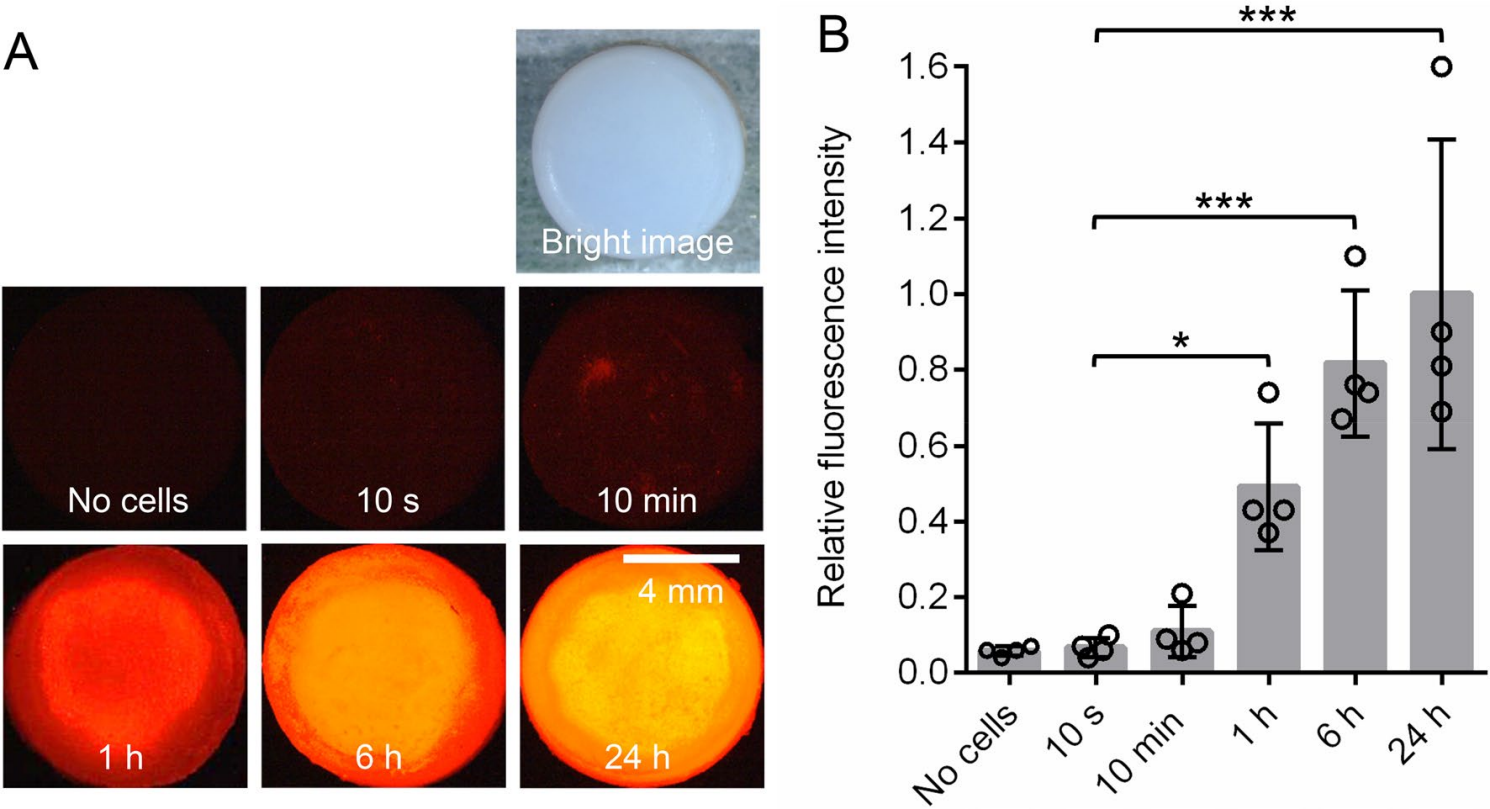
Quantification of adhered MSCs by fluorescence intensity measurements. A DiI-labeled MSC suspension was placed on the porcine abraded cartilage, and the cartilage was washed after a specified time and observed by fluorescence microscopy. (**A**) Fluorescence images. (**B**) Quantification of the fluorescence intensity. Relative intensity is expressed as a ratio to that at 24 h. The number of cylindrical cartilage pieces per MSC donor was 2 or 3. This experiment was repeated four times independently and plotted (*p < 0.05, ***p < 0.001).

The second method involved labeling the MSC suspension with DiI and then measuring the fluorescence intensity. The cartilage showed barely any fluorescence at 10 s and 10 min, but fluorescence was clearly detectable at 1 h and later (Fig. 4A). The relative fluorescence intensity increased to 50% at 1 h, 80% at 6 h, and 100% at 24 h (Fig. 4B).

**Figure 5:**
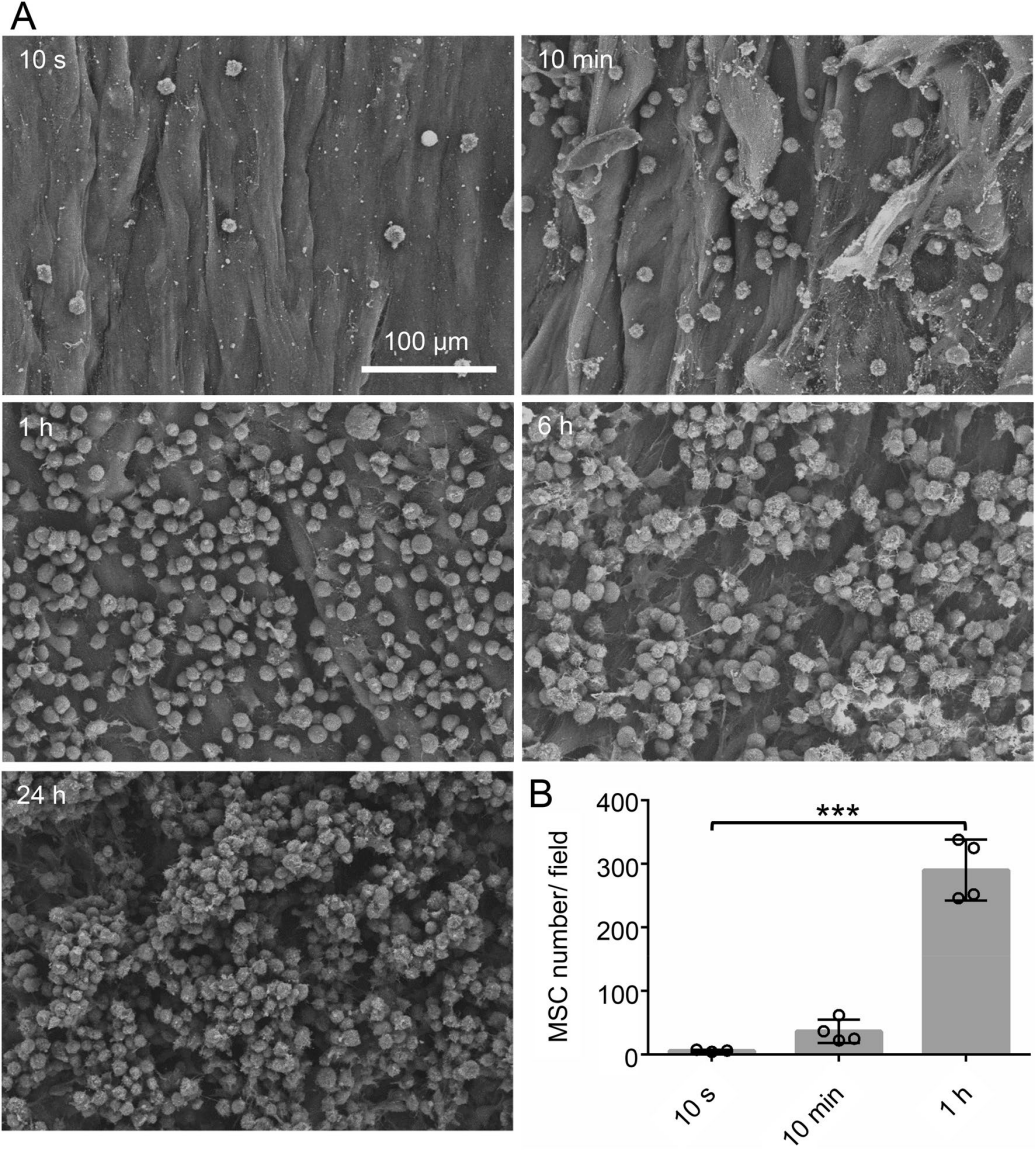
Observation and quantification of adhered MSCs by scanning electron microscopy (SEM). An MSC suspension was placed on the abraded porcine cartilage, and the cartilage was washed after a specified time and observed by SEM. (**A**) SEM images. (**B**) Quantification of adhered MSCs. The number of MSCs in a 320 µm × 380 µm area was counted in 5 or 9 fields per cylindrical cartilage piece. The number of cylindrical cartilage pieces per MSC donor was 2 or 3. This experiment was repeated 3 or 4 times independently and plotted (***p < 0.001).

The third method was direct SEM observation of the synovial MSCs adhered to the cartilage. Some MSCs had already adhered to the cartilage by 10 s (Fig. 5A) and the number of adhered MSCs increased at 10 min and 1 h and time points beyond, but the actual numbers could not be determined at 6 h and 24 h due to cell overlaying. The number of MSCs per field was 6 cells at 10 s, 30 cells at 10 min, and 290 cells at 1 h (Fig. 5B).

### Microspikes and pseudopodia in adhered MSCs

**Figure 6:**
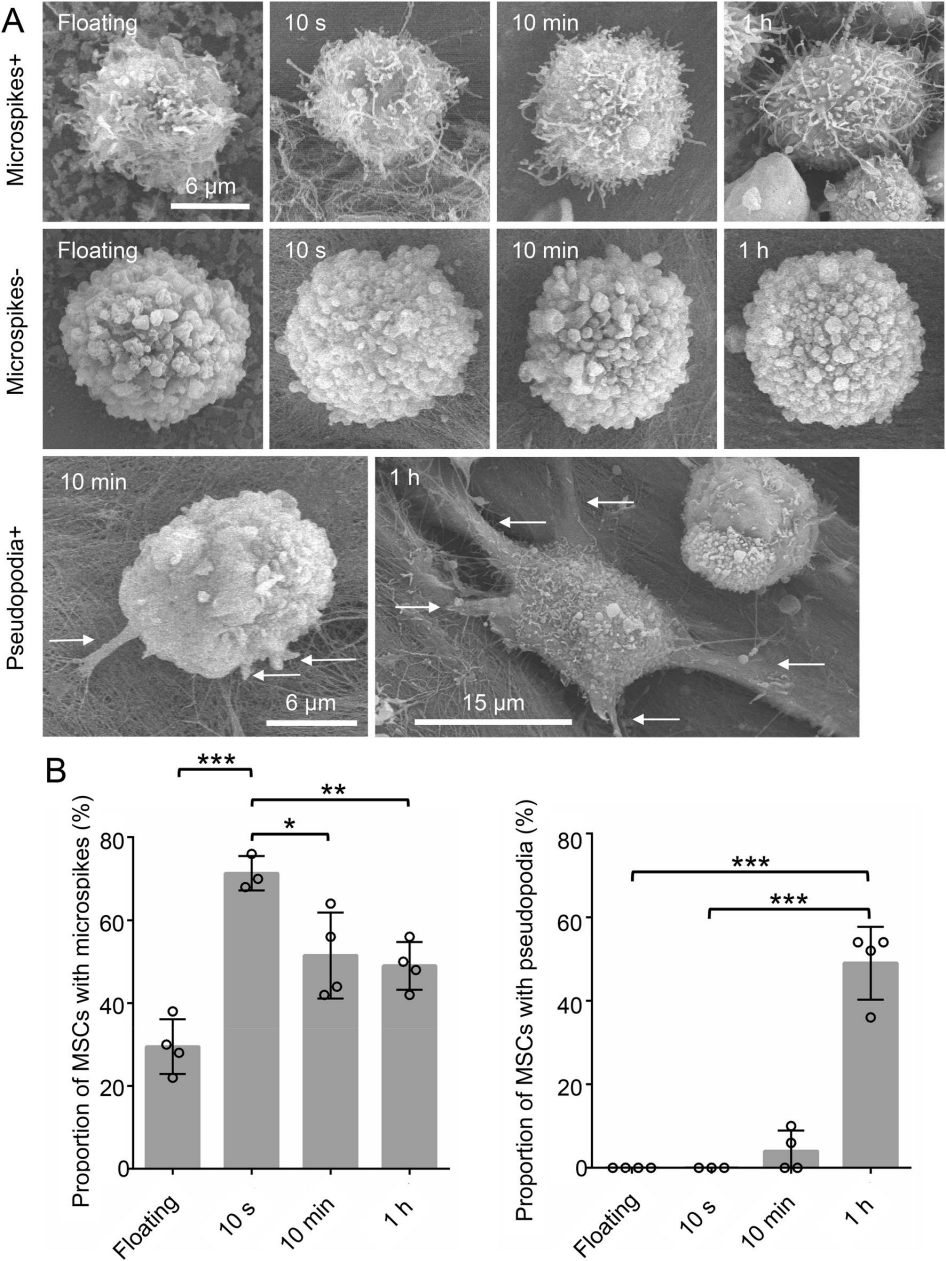
Scanning electron microscopy observation and quantification of microspikes and pseudopodia in adhered MSCs. (**A**) Representative SEM images of MSCs with microspikes and pseudopodia. The pseudopodia are indicated by arrows. (**B**) Quantification of MSCs with microspikes and with pseudopodia. Fifty MSCs were observed per MSC donor. This experiment was repeated 3 or 4 times independently and plotted. Data are also expressed as the average ± standard deviation (*p < 0.05, **p < 0.01, ***p < 0.001).

Synovial MSCs were classified as MSCs with and without microspikes (Fig. 6A). The proportion of MSCs with microspikes was 30% in the original suspension and 71% at 10 s, but the proportion then decreased to 50% at 10 min and remained at 50% at 1 h (Fig. 6B). Synovial MSCs were also classified as MSCs with and without pseudopodia (Fig. 6A). The proportion of MSCs with pseudopodia was 0% in the original suspension and at 10 s, then slightly increased to 4% at 10 min, and significantly increased to 49% at 1 h (Fig. 6B).

### Influence of surface abrasion of porcine cartilage

**Figure 7:**
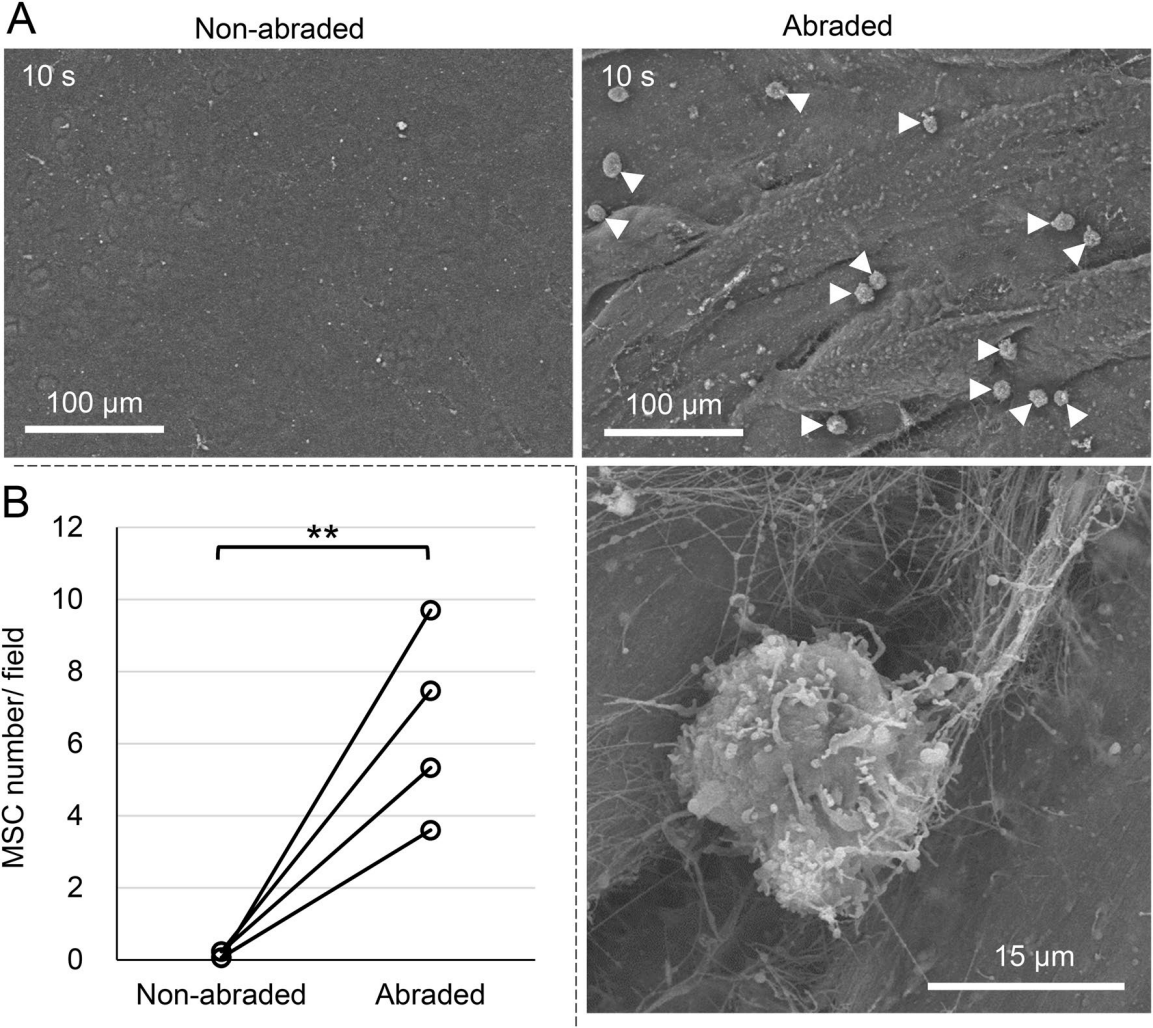
Influence of abrasion of the porcine cartilage surface on MSC adhesion. An MSC suspension was placed on a non-abraded or abraded pig cartilage surface for 10 s, then the cartilage was washed and observed. (**A**) SEM images. Arrowheads indicate adhered MSCs. (**B**) Quantification of adhered MSC. The number of MSCs per 320 µm × 380 µm area was counted in 9 fields per cylindrical cartilage. Three cylindrical cartilage pieces per MSC donor were used. This experiment was repeated 4 times independently and plotted (**p < 0.01).

Few adhered MSCs were observed following application of the MSC suspension for 10 s to a non-abraded surface of pig cartilage, whereas some MSCs were observed on an abraded surface (Fig. 7A). The number of MSCs was 0.1 ± 0.1 per field on the non-abraded cartilage and 6.5 ± 2.6 on the abraded cartilage (*p* < 0.05) (Fig. 7B). High magnification SEM images confirmed that the microspikes on the surfaces of synovial MSCs became entangled in the collagen fibers of the abraded cartilage (Fig. 7A).

## Discussion

The cells derived from the synovium of OA patients formed cell colonies after 14 days of culture. The colony-forming cells were positive for CD44, 73, 90, and 105 and negative for CD45. The cells differentiated into chondrocytes and adipocytes and calcified when cultured in differentiation media. These data indicated that the cells we prepared had characteristics of MSCs^21^.

The surface of the abraded porcine cartilage demonstrated a sawtooth-like structure at low SEM magnification and intertwined collagen fibers at higher magnification. Clark et al. reported numerous fissures among the collagen fibers in the most superficial region of degenerated cartilage, and noted exposure of the free ends of radial collagen fibers in SEM images^17^. The human OA cartilage we examined also exhibited these features, and abrasion of porcine cartilage induced morphological changes similar to those observed in human OA cartilage.

We used three different methods to quantify the proportion/number of synovial MSCs that adhered to abraded porcine cartilage. The first method, which involved determining the proportion of adhered MSCs by counting the numbers of non-adhered MSCs in the washing fluid, indicated that 28% of the initially applied MSCs had adhered at 10 s after placement, and this proportion increased with time. The advantage of this counting method is that measurements can be performed at times ranging from 10 s to 24 h. The disadvantage is that the adhered MSCs were not counted directly, so the reported proportion might be higher than the actual number if some MSCs adhered to the sides of the cartilage cylinder. We previously used a similar method to determine the proportion of synovial MSCs that adhered to abraded porcine meniscus^22^. The proportion of MSCs that adhered to the meniscus was 30% immediately after the placement, increased with time, and reached 96% at 24 h. A similar time course run in the present study indicated that the proportion of synovial MSCs that adhered to the cartilage was comparable to that adhered to the meniscus in our previous study.

The second method was to measure the fluorescence intensity after applying DiI-labeled MSCs to the cartilage. The fluorescence intensity showed a time-dependent increase from 1 to 24 h after cell placement. This method was useful for measurements from 1 to 24 h, but its sensitivity was too low for detection of adhered MSCs at times less than 10 min.

The third method was direct counting of adhered MSCs observed in SEM images. Adherent MSCs were already observed at 10 s after placement, and the number of adherent MSCs increased with time. This method appeared to be the most sensitive and reliable for determining the number of adhered MSCs for times up to 1 h. The disadvantage is that the cell numbers cannot be determined beyond 6 h because the MSCs begin to overlap. Overall, each of these methods for quantifying the proportion/number of adherent synovial MSCs had its own specific strengths and weaknesses.

Microspikes were present in 30% of the synovial MSCs in the original suspension but were found in 70% of the MSCs that had adhered at 10 s. Microspikes are slender cytoplasmic projections that extend from migrating MSCs and have roles in migration, sensing, and cell–cell adhesion^23^. Our SEM observations suggested that microspikes play an adhesive role by entangling with the collagen fibers of the abraded cartilage. In our previous study, microspikes also played an important role in the initial adhesion of synovial MSCs onto an injured meniscus^22^. These findings indicate that microspikes can catch onto collagen fibers on the surfaces of degenerated cartilage and menisci for initial adhesion.

Pseudopodia in synovial MSCs were seldom observed within 10 min of MSC application but were clearly present in 50% of the MSCs at 1 h. Pseudopodia have an important role in the formation of transient adhesions during migration^24,25^. In our previous meniscus study, the proportion of MSCs with pseudopodia also increased from 0 to 54% in 1 h^22^. We suspect that once the synovial MSCs are physically trapped in the degenerated cartilage, the pseudopodia then participate in further adhesion.

Synovial MSCs barely attached to non-abraded pig cartilage. The surface of this cartilage remained smooth and showed no fraying or tangling of collagen fibers. This suggests that no collagen fibers would be available for microspike entrapment, and the pseudopodia would not function effectively.

The findings presented here have clinical relevance, as they raise two interesting prospects for the treatment of cartilage defects using MSC suspensions^26^. One is that the current strategy of creating a full cartilage defect by removing the remaining cartilage down to the subchondral bone may not be necessary for cartilage repairs. If MSCs can be attached to cartilage with partial thickness injury, the transplanted cells can produce trophic factors on the remaining cartilage and/or produce cartilage matrix on their own, thereby promoting cartilage regeneration.

A second idea is that strategies that promote the generation of MSCs with microspikes could increase the efficiency of adhesion and provide a better clinical outcome even with the same volume of MSC suspension containing the same number of MSCs as used in this study. If the surface antigens associated with microspikes are identified, this can be used to sort MSCs to prepare a high rate of cells with microspikes. Shurer et al. reported that glycocalyx on the plasma membrane expresses tubular structures like microspikes by regulating plasma membrane morphology through their length and density on the plasma membrane^27^. This is also expected to be a strategy to promote the generation of MSCs with microspikes.

We used synovial MSCs derived from synovial tissue in the current study. MSCs can be obtained from synovial fluid with a less invasive procedure. The properties of the synovial fluid MSCs, including surface antigens and chondrogenic potential, are similar and the gene expression profiles are comparable, to those of synovial MSCs^28,29^. The most critical disadvantage of synovial-fluid MSCs is that the number of MSCs prepared at passage 0 was much smaller for synovial-fluid MSCs than for synovial MSCs^30,31^. Our aim was to use MSCs with a low number of passages; therefore, we chose synovial MSCs rather than synovial-fluid MSCs.

Our study had four limitations. One was the use of abraded porcine cartilage as degenerated cartilage for the experiments, rather than human OA cartilage. This choice was made because homogeneity cannot be ensured with cartilage obtained from human OA samples. The surface of human OA cartilage will not always reflect that of experimentally abraded porcine cartilage, but it could be more closely approximated if the most superficial layer of the remaining human OA cartilage is abraded prior to MSC therapy. The second limitation is that abrasion of cartilage does not strictly reflect the pathophysiology of OA. Degenerative cartilage that more closely represents OA in humans should be prepared by harvesting OA cartilage induced by surgical procedures or drug injections in animal models. The third limitation is that this study was an ex vivo study, so it does not fully reflect the biological environment. Some important factors, such as joint fluid containing hyaluronic acid and joint movement, cannot be reproduced ex vivo. The fourth limitation is that we did not calculate the viability of the MSCs that adhered to the damaged cartilage. We counted the number of viable cells in the floating MSCs and confirmed that almost all were viable; nevertheless, we cannot rule out the possibility that some cells died after they adhered to the damaged cartilage.

In conclusion, MSCs adhered to degenerated cartilage after placement of an MSC suspension onto the cartilage surface. Three independent quantification methods demonstrated a time-dependent increase in the proportion/number of MSCs that adhered between 10 s and 24 h. MSCs that adhered between 10 s and 1 h had a higher proportion of microspikes, whereas MSCs that adhered after 1 h had a higher proportion of pseudopodia.

## Methods

### Isolation of human synovial MSCs

**Figure 8:**
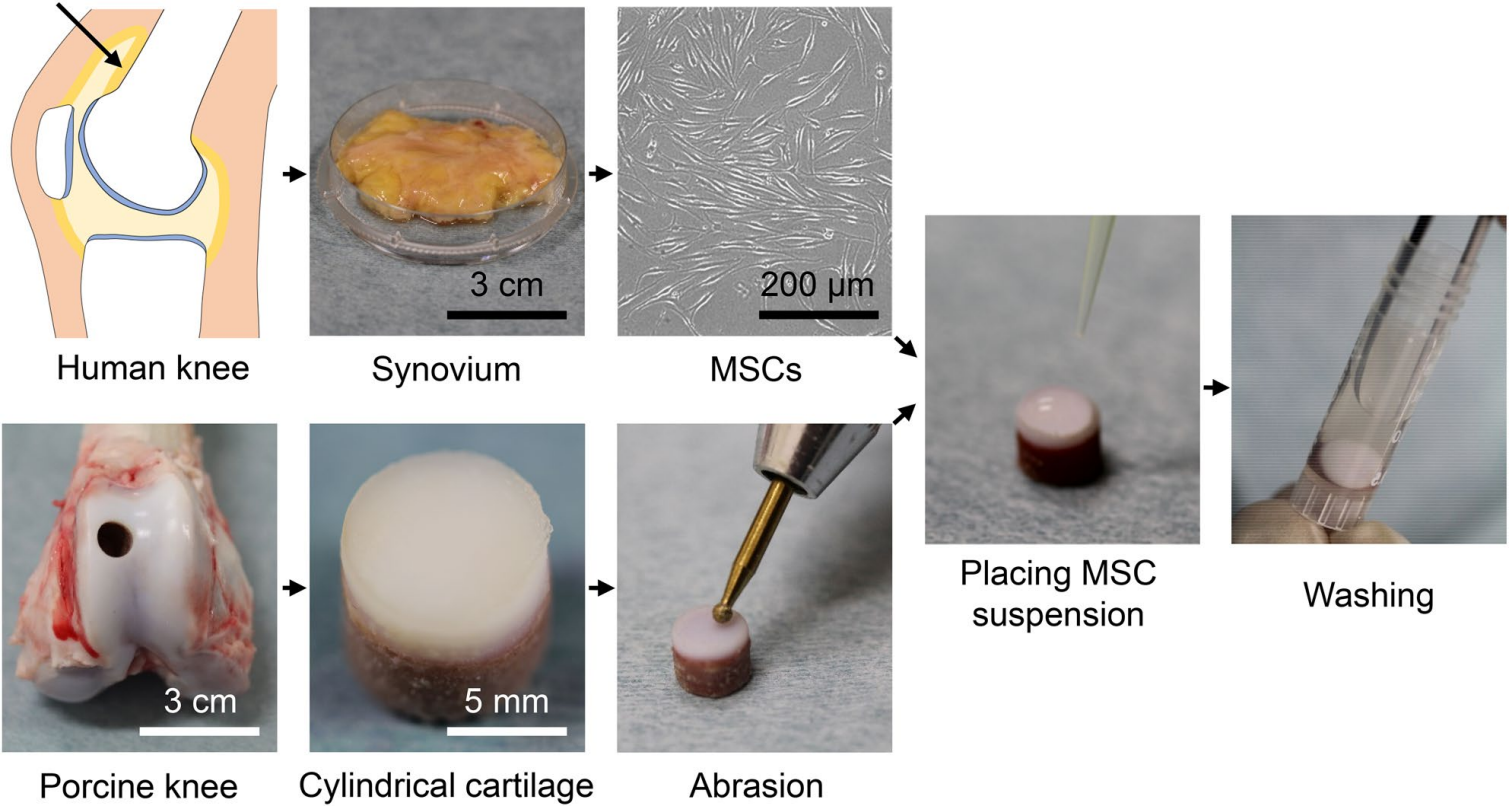
Scheme of the experiment. Mesenchymal stem cells (MSCs) were isolated from the synovium of the knee of a human patient with osteoarthritis. A cylindrical piece of cartilage was removed from the femur of a pig and its surface was abraded. The MSC suspension was placed on the cartilage surface, washed after a specified time, and the number and morphology of MSCs adhered to the surface of the cartilage were evaluated.

The procedures involved human cells were performed in accordance with the standards of the Declaration of Helsinki (1989) and approved by the Medical Research Ethics Committee of Tokyo Medical and Dental University. Written informed consent was obtained from all study subjects. Human synovium was harvested from the knees of eight OA patients (mean age 75 ± 5 years; 4 males and 4 females) during total knee arthroplasty operations (Fig. 8). The synovium was minced and digested in a solution of 3 mg/mL collagenase (Sigma-Aldrich, St Louis, MO, USA) at 37 °C for 3 h, and the digest was filtered through a 70 µm cell strainer (Greiner Bio-One GmbH, Frickenhausen, Germany). The obtained nucleated cells were cultured in a growth medium consisting of α-MEM (Thermo Fisher Scientific, Rockford, IL, USA), 1% antibiotic–antimycotic (Thermo Fisher Scientific), and 10% fetal bovine serum (FBS, Thermo Fisher Scientific) at 37 °C in 5% CO_2_ for 14 days. The resulting human synovial MSCs were harvested at passage 0 and stocked in 95% growth medium and 5% dimethyl sulfoxide (DMSO; Fujifilm Wako Pure Chemical Corporation, Osaka, Japan). Colony formation assays were performed by culturing 100 MSCs in a 60 cm^2^ dish for 14 days, followed by staining with crystal violet (Fujifilm Wako Pure Chemical Corporation).

### Differentiation assays

For chondrogenesis, 2.5 × 10^5^ human synovial MSCs were suspended in 0.5 mL chondrogenic induction medium consisting of DMEM (Sigma-Aldrich) supplemented with 10 ng/mL transforming growth factor-β3, (Miltenyi Biotec, Bergisch, Germany), 500 ng/mL bone morphogenetic protein-2 (Medtronic, Minneapolis, MN, USA), 40 µg/mL proline (Sigma-Aldrich), 100 nM dexamethasone (Fujifilm Wako Pure Chemical Corporation), 100 µg/mL pyruvate (Sigma-Aldrich), 50 µg/mL ascorbic acid 2-phosphate (Fujifilm Wako Pure Chemical Corporation), and 1% ITS Premix (Corning, NY, USA). The MSCs were pelleted by centrifugation at 1500 rpm for 10 min and then cultured for 21 days. The pellets were sectioned and stained with safranin O (Fujifilm Wako Pure Chemical Corporation).

For adipogenesis, 100 synovial MSCs were cultured in growth medium in a 60 cm^2^ dish for 14 days to produce cell colonies. The adhered MSCs were cultured for a further 21 days in an adipogenic induction medium consisting of growth medium supplemented with 100 nM dexamethasone (Fujifilm Wako Pure Chemical Corporation), 0.5 mM isobutylmethylxanthine (Sigma-Aldrich), and 100 µM indomethacin (Sigma-Aldrich). Lipid droplets were stained with oil red O (Muto Pure Chemicals, Tokyo, Japan).

For calcification, 100 synovial MSCs were cultured in growth medium in a 60 cm^2^ dish for 14 days to produce cell colonies. The adhered MSCs were cultured for a further 21 days in a calcification induction medium consisting of growth medium supplemented with 50 µg/mL ascorbic acid 2-phosphate, 1 nM dexamethasone, and 10 mM β-glycerophosphate (Sigma-Aldrich). Calcification was assessed by alizarin red staining (Merck Millipore, Billerica, MA, USA).

### Flow cytometry

Human synovial MSCs were detached with TrypLE (Thermo Fisher Scientific) and suspended in phosphate buffered saline (PBS) supplemented with 2% FBS and 5 mM ethylenediaminetetraacetic acid (EDTA) (Dojindo, Kumamoto, Japan) at a density of 5 × 10^5^ MSCs/mL. The MSCs were stained for 30 min with antibodies against the following antigens: CD44 (PE-Cy7), CD45 (APC-H7), CD73 (V450), CD90 (PE), and CD105 (APC) (all from Becton, Dickinson and Company; BD, NJ, USA). Cell fluorescence was evaluated with a FACSVerse instrument (BD). Data were analyzed using FlowJo software (Tree Star Software, CA, USA).

### Degenerated cartilage

Fresh porcine knees from 6-month-old animals were purchased from Shibaura Zoki Co., Ltd (Tokyo, Japan), and the femoral bones were excised. The femoral groove cartilage with subchondral bone was hollowed out into a cylindrical shape (diameter: 8 mm, height: 5 mm) with a hole saw (Fig. 8). The surface of the cartilage was abraded with an air drill to reproduce a 50% partial-thickness injury. Abraded and non-abraded cartilage were viewed and compared after India ink staining.

### Human OA cartilage

Human knee joint cartilage was harvested from one donor (a 77-year-old male) with OA who underwent a total knee arthroplasty operation. Macroscopically damaged cartilage on the medial side of the tibial plateau was hollowed out.

### Scanning electron microscopy (SEM)

The cartilage was fixed in 2.5% glutaraldehyde in 0.1 M phosphate buffered saline (PBS) for 2 h and washed overnight in 0.1 M PBS at 4 °C. The specimens were then postfixed with 1% osmium tetroxide for 2 h at 4 °C and dehydrated in graded ethanol solutions. After exchanging with 3-methyl butyl acetate and critical point drying, the specimens were coated with platinum. The surface was observed by SEM (S-4500; Hitachi Ltd., Tokyo, Japan).

### Adhesion of human synovial MSCs to the cartilage surface

Stocked human synovial MSCs at passage 0 were thawed and cultured for 14 days in growth medium in a 145 cm^2^ dish at a cell density of 100 MSCs/cm^2^ (Fig. 8). The MSCs were detached with trypsin–EDTA (Thermo Fisher Scientific), and suspended in PBS. The cell suspension, consisting of 10^6^ synovial MSCs in 50 µL PBS was placed on the surface of the cartilage. After 10 s, 10 min, or 1, 6, or 24 h, the cartilage was washed with 10 dips in 950 µL PBS.

### Counting of non-adhered MSCs

The number of non-adhered MSCs in the washes was determined with a LUNA™ Automated Cell Counter (Logos Biosystem, VA, USA). The proportion of MSCs adhered to the cartilage was calculated indirectly by counting the non-adhered MSCs and subtracting that value from the original cell count.

### Fluorescence intensity measurements

Synovial MSCs were labeled with DiI (Thermo Fisher Scientific) and then placed onto the abraded cartilage surface. The cartilage was washed with PBS, and the adhered MSCs were observed with a fluorescence microscope (Leica, Wetzlar, Germany). The relative fluorescence intensity of each cartilage model was calculated using the “Measure” plug-in for Image J (National Institute of Health, MD, USA).

### MSC counts by scanning electron microscopy

SEM images of 5 to 9 fields of view (320 × 380 µm) per one cartilage model were randomly selected. The number of human synovial MSCs in the fields was counted manually, and the average was calculated.

### Evaluation of microspikes and pseudopodia

Synovial MSCs containing microspikes and pseudopodia were quantified from SEM images of 50 randomly selected MSCs from each donor. The selected MSCs were classified by the presence or absence of microspikes and pseudopodia. Microspike-positive MSCs were defined as MSCs with at least three microspikes, according to a previous study^22^. Pseudopodia-positive MSCs were defined as MSCs with at least one pseudopodium. The MSCs in the original suspension were evaluated similarly.

### Influence of surface abrasion on cell adhesion

A cell suspension containing 10^6^ synovial MSCs in 50 µL PBS was placed on the surface of abraded or non-abraded cartilage. After 10 s, the cartilage was washed with PBS. Using SEM, 9 fields of view (320 × 380 µm) per one cartilage model were randomly selected. The numbers of MSCs in each field were counted manually, and the average was calculated.

### Statistical analysis

All statistical analyses were conducted with GraphPad Prism 6 (GraphPad Software, CA, USA). Comparisons between two groups were made using an unpaired t-test. Other data were evaluated by one-way ANOVA, followed by Holm-Sidak’s multiple comparison test. Data were expressed as average ± standard deviation. *P* values < 0.05 were considered statistically significant.

### Ethics declarations

This study was approved by the Medical Research Ethics Committee of Tokyo Medical and Dental University (approval No. M2017-142), and informed consent was obtained from all study subjects.

## Data Availability

The datasets used and/or analyzed during the current study are available from the corresponding author on reasonable request.

## Abbreviations

DMEM: Dulbecco’s Modified Eagle Medium
DMSO: Dimethyl sulfoxide
EDTA: Ethylenediaminetetraacetic acid
FBS: Fetal bovine serum
MEM: Minimum essential medium
MSC: Mesenchymal stem cell
OA: Osteoarthritis
PBS: Phosphate buffered saline
SEM: Scanning electron microscope
